# Treatment Dynamics in People Who Initiate Metformin or Sulfonylureas for Type 2 Diabetes: A National Cohort Study

**DOI:** 10.3389/fphar.2021.794273

**Published:** 2021-12-14

**Authors:** Stephen Wood, Dianna J. Magliano, J Simon Bell, Jonathan E. Shaw, Jenni Ilomäki

**Affiliations:** ^1^ Centre for Medicine Use and Safety, Faculty of Pharmacy and Pharmaceutical Sciences, Monash University, Melbourne, VIC, Australia; ^2^ Baker Heart and Diabetes Institute, Melbourne, VIC, Australia; ^3^ School of Public Health and Preventive Medicine, Monash University, Melbourne, VIC, Australia

**Keywords:** pharmacoepidemiology, antihyperglycaemic agent, addition, switch, type 2 diabetes

## Abstract

**Aim:** To investigate the incidence of, and factors associated with addition and switching of glucose-lowering medications within 12-months of initiating metformin or a sulfonylurea for type 2 diabetes (T2D).

**Methods:** We identified 109,573 individuals aged 18–99 years who initiated metformin or a sulfonylurea between July 2013 and April 2015 using Australian National Diabetes Service Scheme (NDSS) data linked with national dispensing data. Cox proportional hazards regression was used to estimate adjusted hazard ratios (HRs) with 95% confidence intervals (CI) for factors associated with time to addition to or switch from metformin or sulfonylurea over a 12-months follow-up.

**Results:** Treatment addition or switching occurred in 18% and 4% of individuals who initiated metformin and in 28% and 13% of individuals who initiated sulfonylureas. Median time to addition was 104 days for metformin and 82 days for sulfonylureas. Median time to switching was 63 days for metformin and 52 days for sulfonylureas. Congestive heart failure, nicotine dependence, end stage renal disease and dispensing of systemic corticosteroids were associated with higher likelihood of treatment additions and switching in individuals initiating metformin. Antipsychotic dispensing was associated with a higher likelihood of treatment addition in individuals initiating sulfonylureas. Women initiating metformin were less likely to receive treatment additions but more likely to switch treatment than men.

**Conclusion:** Nearly one quarter of Australians who initiate treatment for T2D with metformin or sulfonylureas switch or receive additional treatment within 12-months, with those who initiate sulfonylureas more likely to switch or receive additional treatment than those who initiate metformin.

## Introduction

Type 2 diabetes (T2D) is a progressive disease which often requires treatments to be added or switched in order to achieve glycated haemoglobin (HbA_1C_) targets. Australian guidelines recommend adding a T2D medication when individuals have failed to reach glycaemic targets after 3–6 months of metformin or sulfonylurea monotherapy (Royal Australian College of General Practitioners, 2020). Similar treatment recommendations are included in international guidelines (Cornell, 2017; American Diabetes Association, 2020). Our previous research has demonstrated 90% of Australians initiate medication treatment with either metformin or sulfonylurea monotherapy (Wood et al., 2020).

People with T2D and other cardiovascular risk factors may benefit from more aggressive treatment for hyperglycaemia (Royal Australian College of General Practitioners, 2020). However, the median time to treatment intensification after a high HbA_1C_ reading is greater than 1 year (Khunti et al., 2018). Pantalone et al. reported that 44.4% of individuals with an HbA_1C_ ≥9.0% (75 mmol/mol) did not receive treatment intensification within 6 months (Pantalone et al., 2018). Paul et al. found that delaying treatment intensification beyond 1 year increases the risk of myocardial infarction (MI), heart failure (HF), stroke and composite cardiovascular events (Paul et al., 2015).

Early, aggressive treatment is important in younger people due to the elevated risk of premature death from cardiovascular disease (CVD) (Huo et al., 2018). However, stringent glycaemic targets in people aged >65 years may increase the risk of hypoglycaemia (American Diabetes Association, 2018). People with multimorbidity may be less likely to receive multiple T2D therapies due to concerns about polypharmacy and drug interactions. T2D medication may be switched due to lack of efficacy or adverse drug events (ADEs). An Irish study reported that sulfonylurea initiators were more likely than metformin initiators to receive a treatment addition or switch within 2 years (Grimes et al., 2015). Our study is the first in Australia to distinguish between treatment addition and switching in T2D. The objective of this study was to investigate the incidence of, and factors associated with switching and addition of glucose-lowering medications within 12-months of initiating metformin or a sulfonylurea for T2D.

## Materials and Methods

### Study Design, Data Source and Study Population

We conducted a national population-based cohort study on the incidence of, and factors associated with T2D medication addition and switching between July 2013 and April 2016. We utilised data from the Australian National Diabetes Services Scheme (NDSS) linked to national pharmacy dispensing data from Australia’s Pharmaceutical Benefits Scheme (PBS). Linkage of NDSS and PBS data was performed by the Australian Institute of Health and Welfare (AIHW) for the period of January 2002 to April 2016.

The NDSS provides education and subsidies for 80–90% of Australians diagnosed with diabetes (Huo et al., 2018). NDSS registration is performed by a medical practitioner or certified diabetes educator (Huo et al., 2018). NDSS data include each registrant’s date of birth, date of diabetes diagnosis, postcode and date of death (*via* a linkage to the National Death Index). Socio-Economic Indexes for Areas (SEIFA) score and Accessibility/Remoteness Index of Australia (ARIA) scores were derived from postcodes. SEIFA scores were divided into quintiles (Koye et al., 2019). The ARIA score identifies five area categories; major urban, inner regional, outer regional, remote, and very remote areas based on distance from major service centres (Koye et al., 2019). In our study, the remote and very remote categories (collectively, 2% of the population) were collapsed into one category (Koye et al., 2019).

The PBS entitles Australia’s 25 million citizens, permanent residents and people from countries with reciprocal health care agreements to receive government-subsidised medications. PBS data include medication name and strength, dispensed quantity, date of prescribing and date of supply. PBS reimbursement criteria require people to trial metformin or a sulfonylurea before other T2D medications.

**FIGURE 1:**
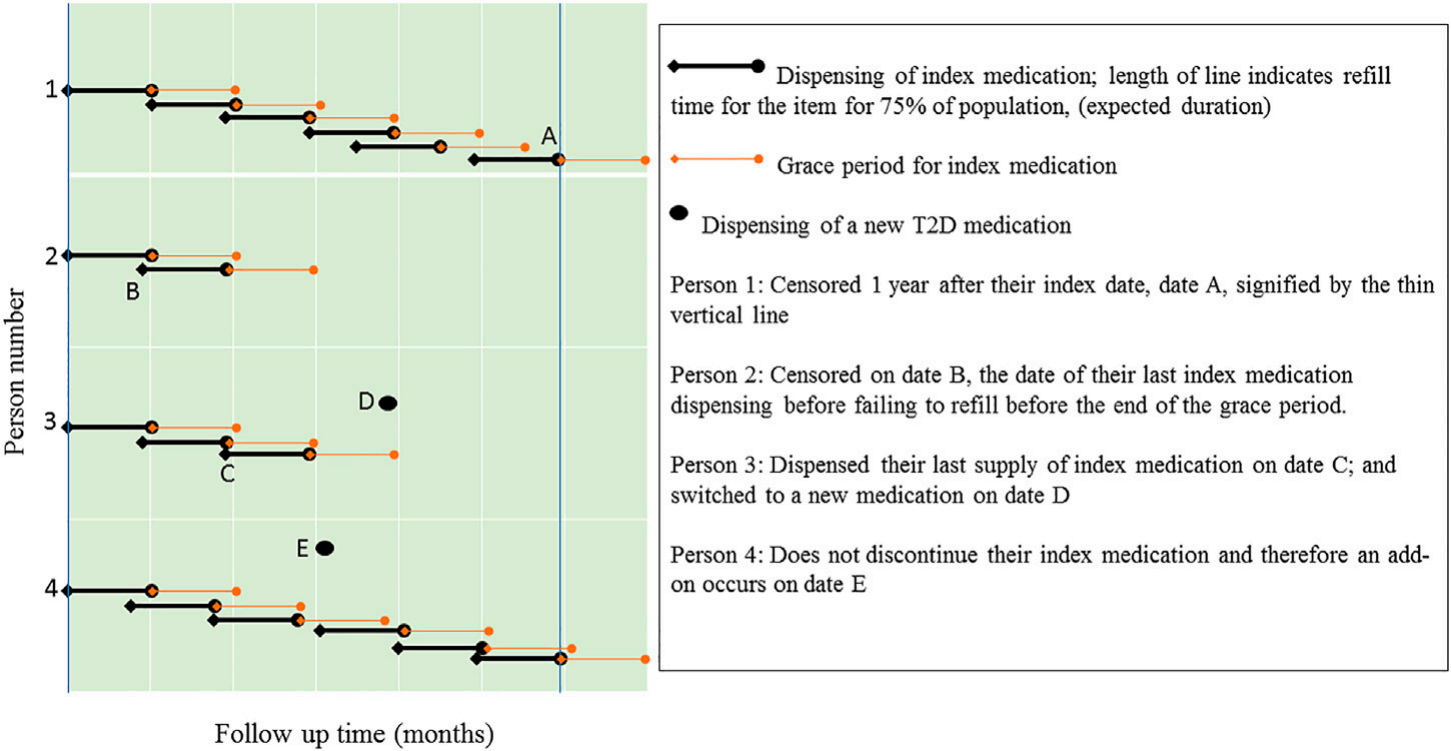
Illustration through examples how additions and switches were identified.

The study population included all adults aged 18–99 years diagnosed with T2D who initiated metformin or a sulfonylurea (the index medication) between July 1, 2013, and April 30, 2015. The index date was the date of first dispensing of either metformin or sulfonylurea with no dispensings of any diabetes medications in the previous 12 months (Figure 1). We excluded individuals dispensed more than one T2D medication on their index date or with a recorded date of death on or prior to their index date.

### Measures and Definitions

Metformin (A10BA) and sulfonylureas (A10BB) were categorised using the Anatomical Therapeutic Chemical (ATC) classification system (World Health Organisation, 2011). Sulfonylureas included glibenclamide, gliclazide, glimepiride and glipizide.

The Rx-Risk Index was used to identify each person’s comorbidities based on medication dispensing. The index has been validated for use with Australian PBS data (Pratt et al., 2018). All people had T2D, so we deducted 1 from each individual’s comorbidity score. We also used the Rx-Risk Index to infer specific comorbidities during the year prior to the index date. These comorbidities included congestive heart failure (CHF), hyperlipidaemia, depression, nicotine dependence, hypertension, and end stage renal disease (Supplementary Appendix S1). Depression is known to be associated with poor adherence (Caughey et al., 2013) and tobacco smoking with cardiovascular risk (Banks et al., 2019). Dispensings of systemic corticosteroids (ATC code H02A) or antipsychotics (N05A) in the 3 months prior to the index date were included in the model because these medications may affect glycaemic control. Other potential factors we investigated were age, socioeconomic status (SEIFA), remoteness/rurality (ARIA), sex, Aboriginal or Torres Strait Islander status and time between T2D diagnosis and the index date. The date of diabetes diagnosis was missing for 16% of individuals and for these individuals we used the date of the NDSS enrolment as a proxy for date of diagnosis.

### Outcome Measures

Medication addition or switching was defined as dispensing of a T2D medication other than the index medication, including metformin, sulfonylurea, acarbose (A10BF), thiazolidinedione (A10BG), dipeptidyl peptidase-IV inhibitor (DPP-4I; A10BH) glucagon-like peptide-1 agonist (GLP-1A; A10BJ), sodium-glucose cotransporter-2 inhibitor (SGLT-2Is; A10BK and A10BX), fixed dose combination therapy (A10BD) or insulin (A10A). Insulins included all available insulin products (fast acting, intermediate acting long acting and mixed insulin and insulin analogues for injection or inhalation) (World Health Organisation, 2011).

**FIGURE 2:**
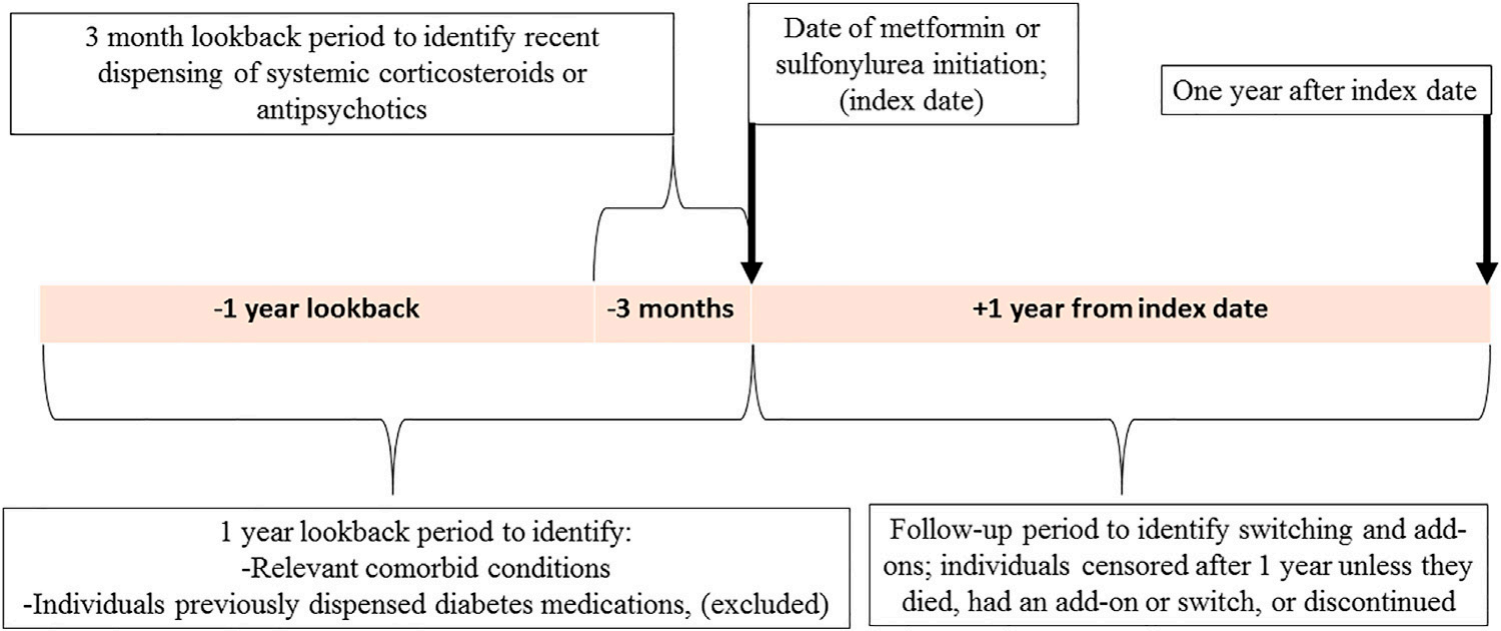
An illustration depicting the study design.

The duration of each prescription was estimated using the prescription refill period. The duration of a specific PBS medication was defined as the period in which 75% of the population refilled their prescription for that item (Vitry et al., 2010). If an individual did not refill the index medication before the end of the grace period for the previous supply, the individual was deemed to have discontinued the index medication. An addition was defined as dispensing of a new T2D medication without discontinuing the index medication. A switch was defined as dispensing of a new T2D medication after the last dispensing of a discontinued index medication (Figure 2). When investigating additions of medications, people were censored on the date of switching, death date or 1 year after their index date, whichever occurred first. When investigating switching of medications, people were censored on the date of addition, death date or 1 year after their index date, whichever occurred first.

### Statistical Analysis

Baseline characteristics of the study cohorts were presented as means with standard deviations (SD), medians with interquartile ranges (IQR) or as frequencies and percentages. All analyses were conducted separately for people who initiated metformin and sulfonylurea. The proportional hazards assumption was confirmed, and Cox proportional hazards regression was used to estimate adjusted hazard ratios (HRs) with 95% confidence intervals (CI) for factors associated with time to switching from or addition to initial monotherapy within 365 days. HRs were estimated for age, comorbidity score, SEIFA score, ARIA score, CHF, hyperlipidaemia, depression, nicotine dependence, hypertension, end stage renal disease and the dispensing of antipsychotics or systemic corticosteroids during the previous 3 months.

Sensitivity analyses were performed using different grace periods to define index medication continuation or discontinuation using similar methods to a study by Caughey et al. (2013). We also conducted sensitivity analysis excluding individuals dispensed antipsychotics or systemic corticosteroids during the 3 months prior to the index date to determine if the inclusion of individuals with possible drug-induced T2D may have biased the results towards more aggressive treatment. A third sensitivity analysis (Supplementary Appendix S2) was conducted for individuals dispensed >80% of their prescriptions while eligible for higher PBS reimbursement (concession beneficiaries). Our data was more complete for concession beneficiaries prior to July 2012 and so this provided the opportunity to utilise a two-year lookback period to verify our main analysis successfully captured incident users. All analyses were conducted using the statistical software package SAS version 9.4 (SAS Institute Inc., Cary, NC, United States). This study was approved by the Monash University Human Research Ethics Committee.

## Results

### Cohort Characteristics

Of the 109,573 people in the study cohort, 93.8% initiated metformin and 6.2% initiated a sulfonylurea. The mean ages of people initiating metformin and sulfonylurea therapy were 58.7 (SD 13.2) and 65.7 (SD 14.6) years, respectively, (Table 1). Of the metformin and sulfonylurea initiators, 44.4 and 44.2%, respectively, were women. The respective median numbers of comorbidities in the metformin and sulfonylurea cohorts were 3 (IQR 2–5) and 4 (IQR 2–7). The median time until initiation of the index medication after diagnosis of T2D was 0.2 (IQR 0.0–4.7) years in the metformin initiators and 4.4 (IQR 0.1–9.9) years among sulfonylurea initiators.

**TABLE 1:** Characteristics of metformin and sulfonylurea initiators from the NDSS.

	Metformin initiators	Sulfonylurea initiators	Total
	(*n* = 102,737)	(*n* = 6,836)	(*n* = 109,573)
Age, years (mean ± SD)	58.7 ± 13.2	65.7 ± 14.6	59.2 ± 13.4
18–49	26,195 (25.5)	1,015 (14.8)	27,210 (24.8)
50–74	65,426 (63.7)	3,811 (55.7)	69,237 (63.2)
75–99	11,116 (10.8)	2,010 (29.4)	13,126 (12.0)
Sex, female n(%)^a^	45,634 (44.4)	3,021 (44.2)	48,655 (44.4)
Comorbidity score [median (IQR)]^b^	3 (2–5)	4 (2–7)	3 (2–5)
Number of comorbidities^b^			
0	6,225 (6.1)	399 (5.8)	6,624 (6.0)
1–2	30,042 (29.2)	1,515 (22.2)	31,557 (28.8)
3–4	30,389 (29.6)	1,567 (22.9)	31,956 (29.2)
5+	36,081 (35.1)	3,355 (49.1)	39,436 (36.0)
ARIA score			
1) Major urban	67,853 (66.0)	4,848 (70.9)	72,701 (66.3)
2) Inner regional	22,027 (21.4)	1,171 (17.1)	23,198 (21.2)
3) Outer regional	10,923 (10.6)	610 (8.9)	11,533 (10.5)
4) Remote	1,265 (1.2)	116 (1.7)	1,381 (1.3)
5) Very remote	669 (0.7)	91 (1.3)	760 (0.7)
SEIFA score (mean ± SD)	2.94 ± 1.40	2.98 ± 1.44	2.94 ± 1.40
1. Most disadvantaged	21,300 (20.7)	1,497 (21.9)	22,797 (20.8)
2.	20,589 (20.0)	1,251 (18.3)	21,840 (19.9)
3.	22,782 (22.2)	1,402 (20.5)	24,184 (22.1)
4.	19,057 (18.5)	1,274 (18.6)	20,331 (18.6)
5. Least disadvantaged	19,009 (18.5)	1,412 (20.7)	20,421 (18.6)
Congestive heart failure	5,250 (5.1)	946 (13.8)	6,196 (5.7)
Nicotine dependence	3,189 (3.1)	124 (1.8)	3,313 (3.0)
Depression	23,023 (22.4)	1,420 (20.8)	24,443 (22.3)
Systemic corticosteroids	6,226 (6.1)	884 (12.9)	7,110 (6.5)
Antipsychotics	3,581 (3.5)	231 (3.4)	3,812 (3.5)
Lipid-lowering medications	50,293 (49.0)	3,452 (50.5)	53,745 (49.0)
Hypertension	49,632 (48.3)	3,614 (52.9)	53,246 (48.6)
End stage renal disease	69 (0.1)	250 (3.7)	319 (0.3)
Time between T2D diagnosis and index date, [median±(IQR)], years	0.2 (0.0–4.7)	4.4 (0.1–9.9)	0.3 (0.0–5.0)
Time between T2D diagnosis and index date			
No delay	25,115 (24.4)	966 (14.1)	26,081 (23.8)
<1 year	32,205 (31.3)	1,117 (16.3)	33,322 (30.4)
1–2 years	5,607 (5.5)	351 (5.1)	5,958 (5.4)
>2 years	39,810 (38.7)	4,402 (64.4)	44,212 (40.3)
Aboriginal or Torres Strait Islander status			
Yes	2,995 (2.9)	212 (3.1)	3,207 (2.9)
No	86,829 (84.5)	5,870 (85.9)	92,699 (84.6)
Unspecified	12,913 (12.6)	754 (11.0)	13,667 (12.5)

NDSS National Diabetes Services Scheme; T2D Type 2 Diabetes; ARIA Accessibility/Remoteness Index of Australia; SEIFA Socio-Economic Indexes for Areas; SD Standard deviation; IQR Interquartile Range.

a Unless otherwise stated, figures are quoted as n (%).

b A score of 1 was deducted from the total RxRisk-V score, as the whole cohort had T2D medications prescribed at baseline.

### Incidence of an Addition or Switch

For metformin initiators, the proportions of individuals receiving an addition or switch during the first year were 18 and 4%, respectively, whereas among sulfonylurea initiators the proportions were 28 and 13%, respectively. Overall, 23.2% of the cohort received an addition or a switch. The median time to addition amongst those individuals who received one was 104 days in the metformin cohort and 82 days in the sulfonylurea cohort. The median time to switching amongst those individuals who received one was 63 days in the metformin cohort and 52 days in the sulfonylurea cohort.

### Factors Associated With T2D Treatment Addition or Switch

In both cohorts, there was an inverse association between age and the risk of receiving add-on therapy. In the metformin cohort, compared to people aged 18–49 years, people aged 50–74 (HR 0.77; 95%CI 0.75–0.80) and 75–99 (HR 0.57; 95%CI 0.54–0.61) had lower risks of receiving additions. In the sulfonylurea cohort, compared to people aged 18–49 years, people aged 50–74 (HR 0.70; 95%CI 0.62–0.79) and 75–99 (HR 0.44; 95%CI 0.38–0.52) also had lower risks of receiving add-on therapy, (Table 2). Sulfonylurea initiators aged 50–74 (HR 0.69; 95%CI 0.58–0.82) and 75–99 years (HR 0.42; 95%CI 0.33–0.54) had a lower risk of switching compared with initiators aged 18–49 years.

**TABLE 2:** Factors associated with receiving add-on therapy or treatment switch within one year of starting metformin or sulfonylurea.

	Metformin add-on		Metformin switched		Sulfonylurea add-on		Sulfonylurea switched	
	HR	95%CI	HR	95%CI	HR	95%CI	HR	95%CI
Age, years								
18–49	—	—	—	—	—	—	—	—
50–74	0.77	(0.75–0.80)	0.84	(0.78–0.90)	0.70	(0.62–0.79)	0.69	(0.58–0.82)
75–99	0.57	(0.54–0.61)	1.05	(0.93–1.17)	0.44	(0.38–0.52)	0.42	(0.33–0.54)
Sex, female	0.84	(0.81–0.86)	1.42	(1.33–1.51)	0.98	(0.89–1.07)	1.00	(0.87–1.14)
Number of comorbidities^a^								
0	—	—	—	—	—	—	—	—
1–2	0.87	(0.82–0.92)	1.06	(0.92–1.23)	0.91	(0.76–1.09)	0.95	(0.73–1.24)
3–4	0.80	(0.75–0.85)	1.11	(0.95–1.30)	0.71	(0.58–0.86)	0.84	(0.62–1.13)
5+	0.82	(0.76–0.88)	1.40	(1.18–1.66)	0.53	(0.42–0.66)	0.68	(0.49–0.96)
ARIA score								
1. Major Urban	—	—	—	—	—	—	—	—
2. Inner Regional	0.99	(0.95–1.02)	1.08	(1.00–1.17)	0.95	(0.84–1.09)	1.17	(0.97–1.41)
3. Outer Regional	0.98	(0.93–1.03)	1.02	(0.91–1.13)	0.90	(0.76–1.07)	0.90	(0.70–1.17)
4/5 Remote and very remote	1.00	(0.90–1.11)	0.72	(0.56–0.95)	0.80	(0.59–1.09)	1.00	(0.65–1.52)
SEIFA index								
1. Most Disadvantaged	—	—	—	—	—	—	—	—
2.	1.01	(0.96–1.05)	0.93	(0.85–1.03)	1.12	(0.97–1.29)	0.87	(0.70–1.09)
3.	0.99	(0.95–1.04)	0.90	(0.82–0.98)	1.04	(0.90–1.20)	1.02	(0.83–1.25)
4.	1.02	(0.97–1.07)	0.94	(0.85–1.04)	1.12	(0.97–1.29)	0.97	(0.78–1.20)
5. Least Disadvantaged	0.92	(0.87–0.96)	0.85	(0.77–0.95)	1.15	(1.00–1.33)	1.04	(0.84–1.28)
Congestive Heart Failure	1.29	(1.21–1.38)	1.27	(1.12–1.44)	1.23	(1.06–1.44)	0.91	(0.71–1.16)
Nicotine dependence	1.31	(1.22–1.42)	1.26	(1.07–1.48)	0.98	(0.69–1.38)	1.32	(0.86–2.02)
Depression	1.09	(1.05–1.13)	0.99	(0.91–1.07)	1.03	(0.91–1.17)	1.09	(0.91–1.31)
Systemic corticosteroids	1.15	(1.08–1.22)	1.47	(1.32–1.64)	1.10	(0.94–1.28)	1.26	(1.01–1.56)
Antipsychotics	1.21	(1.13–1.31)	0.91	(0.77–1.08)	1.60	(1.27–2.03)	1.17	(0.81–1.71)
Lipid-lowering medications	0.87	(0.84–0.90)	0.81	(0.75–0.87)	1.02	(0.91–1.13)	0.98	(0.83–1.14)
Hypertension	0.97	(0.93–1.00)	0.86	(0.80–0.92)	1.11	(1.00–1.24)	0.88	(0.75–1.04)
End stage renal disease	1.91	(1.23–2.97)	2.39	(1.19–4.79)	0.75	(0.55–1.02)	0.71	(0.45–1.13)
Time between T2D diagnosis and index date								
No time	—	—	—	—	—	—	—	—
<1 year	0.86	(0.83–0.89)	0.87	(0.80–0.95)	0.80	(0.69–0.92)	0.90	(0.73–1.11)
1–2 years	0.77	(0.71–0.82)	0.84	(0.72–0.99)	0.54	(0.43–0.69)	0.60	(0.43–0.84)
>2 years	0.95	(0.92–0.99)	1.14	(1.05–1.23)	0.62	(0.55–0.69)	0.55	(0.46–0.65)
Aboriginal or Torres Strait Islander status	1.14	(1.06–1.24)	1.11	(0.93–1.33)	0.97	(0.72–1.29)	1.02	(0.68–1.54)

T2D Type 2 Diabetes; ARIA Accessibility/Remoteness Index of Australia; SEIFA Socio-Economic Indexes for Areas; CI confidence interval; HR adjusted hazard ratio.

a A score of 1 was deducted from the total RxRisk-V score, as the whole cohort had T2D medications prescribed at baseline.

Compared with men, women commencing metformin were less likely to receive an add-on medication (HR 0.84; 95%CI 0.81–0.86), but more likely to have their metformin switched (HR 1.42; 95%CI 1.33–1.51). Switching from metformin was also more likely in people with ≥5 (HR 1.40; 95%CI 1.18–1.66) comorbidities compared to those without comorbidities. Sulfonylurea initiators with ≥5 (HR 0.68; 95%CI 0.49–0.96) comorbidities had lower risks of switching compared to those without comorbidities.

In the metformin cohort, CHF (HR 1.29; 95% CI 1.21–1.38), nicotine dependence, (HR 1.31; 95% CI 1.22–1.42), depression (HR 1.09; 95% CI 1.05–1.13), systemic corticosteroids (HR 1.15; 95% CI 1.08–1.22), antipsychotics (HR 1.21; 95% CI 1.13–1.31) and end stage renal disease (HR 1.91; 95% CI 1.23–2.97), were associated with a higher likelihood of receiving add-on therapy. CHF (HR 1.27; 95% CI 1.12–1.44), nicotine dependence, (HR 1.26; 95% CI 1.07–1.48), systemic corticosteroids (HR 1.47; 95% CI 1.32–1.64) and end stage renal disease (HR 2.39; 95% CI 1.19–4.79), were associated with switching from metformin. People initiating metformin who were dispensed lipid-lowering medications were less likely to receive additions (HR 0.87; 95% CI 0.84–0.90), or to switch (HR 0.81; 95% CI 0.75–0.87). In the sulfonylurea cohort, CHF (HR 1.23; 95% CI 1.06–1.44) and antipsychotics (HR 1.60; 95% CI 1.27–2.03) were associated with receiving additional therapy.

Metformin initiators had progressively lower risks of receiving additions to their index medication as time between T2D diagnosis and index date increased from 0–1 year (HR 0.86; 95% CI 0.83–0.89) to 1–2 years (HR 0.77; 95% CI 0.71–0.82) compared to people who received index medication on their T2D diagnosis date. Sulfonylurea initiators with 0–1 year (HR 0.80; 95% CI 0.69–0.92), and 1–2 years (HR 0.54; 95% CI 0.43–0.69) between their T2D diagnosis and index date also had lower risks or receiving additional therapy compared to people who received index medication on their T2D diagnosis date.

### Medications Added or Switched to

The medications most frequently added to metformin were DPP-4Is (48.5%), sulfonylureas (33.0%) and insulin (11.0%), (Table 3). The medications most frequently added to sulfonylureas were metformin (62.7%), DPP-4Is (13.3%), and insulin (12.5%).

**TABLE 3:** Medications added on or switched to during the first year after metformin or sulfonylurea initiation.

	Metformin initiators		Sulfonylurea initiators	
	Added on *N* = 18,522	Switched to *N* = 4,081	Added on *N* = 1,913	Switched to *N* = 863
Dipeptidyl peptidase-4 inhibitor (DPP-4I)	8,984 (48.5)	619 (15.2)	254 (13.3)	90 (10.4)
Sulfonylurea	6,104 (33.0)	2,514 (61.6)	NA	NA
Insulin	2,036 (11.0)	717 (17.6)	239 (12.5)	176 (20.4)
Metformin	NA	NA	1,199 (62.7)	505 (58.5)
Sodium-glucose co-transport inhibitor (SGLT-2I)	987 (5.3)	149 (3.7)	58 (3.0)	15 (1.7)
Glucagon-like peptide-1 agonist (GLP-1A)	349 (1.9)	46 (1.1)	13 (0.7)	5 (0.6)
Fixed-Dose-Combination product (FDC)	NA^a^	NA^a^	127 (6.6)	63 (7.3)
Thiazolidinedione (TZD)	39 (0.2)	20 (0.5)	15 (0.8)	3 (0.3)
Acarbose	23 (0.1)	16 (0.4)	8 (0.4)	6 (0.7)

a All FDC products available during the time of this study contained metformin plus another glucose-lowering medication. When individuals from the metformin cohort commenced an FDC, it was considered an addition/switch with respect to the non-metformin component.

People who switched from metformin were most likely to switch to sulfonylureas (61.6%), insulin (17.6%) or DPP-4Is (15.2%) whereas people switching from a sulfonylurea were most likely to switch to metformin (58.5%), insulin (20.4%) or DPP-4Is (10.4%).

### Sensitivity Analyses

In the first sensitivity analysis, a small number of people (0.7% of people in the metformin cohort and 0.9% of the sulfonylurea cohort), were reclassified as having received add-on therapy where they were previously classified as having switched; however, it did not result in any significant changes to our results in the multivariate models. There were also no substantial changes to our results when we excluded people who received antipsychotic medication or systemic corticosteroids during the 3 months prior to their index date. Supplementary Appendix S2 shows the results obtained when we repeated the analysis in a concessional population with a two-year lookback period, which were generally similar to the main analysis but contained wider confidence intervals due to the smaller population size.

## Discussion

The main finding of this national study was 23.2% of individuals who initiated metformin or a sulfonylurea either switched or received additional treatment within 12-months. Our results were similar to those of an Irish study which reported 35% of metformin and sulfonylurea initiators changed regimens within 2 years (Grimes et al., 2015). Our results also showed that people who initiated sulfonylureas were more likely to switch or receive additional treatment than those who initiate metformin.

Higher rates of treatment switching and addition in people who initiate sulfonylureas may reflect poorer glycaemic control or a higher incidence of ADEs (Sola et al., 2015). Although sulfonylureas lower blood glucose to a greater extent than metformin, both metformin and sulfonylureas have similar effectiveness in achieving target HbA_1C_ (Hemmingsen et al., 2014). However, sulfonylureas have a less favourable ADE profile including weight gain and the risk of hypoglycaemia (Hemmingsen et al., 2014). Current Australian and international guidelines recommend SGLT-2Is and GLP-1As in preference to sulfonylurea monotherapy in people with heart failure or chronic renal disease (American Diabetes Association, 2020; Royal Australian College of General Practitioners, 2020). However, this recommendation was not included in the 2014–15 guidelines (Royal Australian College of General Practitioners and Diabetes Australia, 2014). For this reason, the higher rates of treatment additions and switches in those who initiated sulfonylureas were unlikely to be explained by prescriber adherence to clinical practice guidelines.

Older people who initiated sulfonylureas were less likely to switch than younger people. Older people have a higher prevalence of renal impairment and therefore, few other glucose-lowering medication alternatives. International guidelines (Royal Australian College of General Practitioners and Diabetes Australia, 2014; American Diabetes Association, 2020; Górriz et al., 2020; Royal Australian College of General Practitioners, 2020) state that SGLT-2Is and the GLP-1A exenatide are contraindicated in individuals with a creatinine clearance <30 ml/min/1.73 m^2^. It is recommended that metformin be used with caution in people with mild to moderate renal impairment (Aschenbrenner, 2016). This may have contributed to a lower rate of metformin initiation and to clinical inertia. In 2016 US Food and Drug Administration (FDA) advised that metformin is safe to use in people with mild to moderate renal impairment, acknowledging that the risk of lactic acidosis had been overstated (Aschenbrenner, 2016). Older age was associated with longer time to addition in both sulfonylurea and metformin initiators, possibly reflecting more conservative prescribing for older adults in whom stringent glycaemic control is not recommended (American Diabetes Association, 2018; Royal Australian College of General Practitioners, 2020). Chronic kidney disease is more common in people with multimorbidity (Lee et al., 2018). This may explain why metformin initiators with 5 or more comorbidities had a 40% higher risk of switching compared to those without comorbidities. Moreover, metformin initiators with end stage renal disease had 2.4 times the risk of switching, compared to those without it.

Longer time to treatment switching and addition was observed among people dispensed lipid-lowering medications. A higher proportion of these individuals may have had cardiovascular disease in whom HbA_1C_ targets are likely to be less stringent (Royal Australian College of General Practitioners, 2020). Another explanation is that these people had poor glycaemic control linked to statins (Thakker et al., 2016). However, statin use has only been associated with modest glycaemic changes (Erqou et al., 2014). Conversely, people with CHF had a higher risk of receiving add-on therapy. This may be because co-existing T2D and CHF are associated with increased mortality compared to either condition alone (Swedberg and Rydén, 2016) and a 25% increased risk of cardiovascular death or heart failure hospitalization after 34 months for every 1% increase in HbA_1C_ level (Gerstein et al., 2008). Smoking cessation attempts were associated with receiving add-on therapy and switching. A cohort study by Lycett et al. found smoking cessation was independently associated with deterioration in glycaemic control lasting for 3 years (Lycett et al., 2015). This may explain the higher rate of addition and switching among individuals dispensed smoking cessation products. It may also reflect more intensive diabetes management in people who smoke.

Time between diabetes diagnosis and treatment initiation was associated with treatment addition. Compared to individuals dispensed their index T2D medication on their diagnosis date, people with time intervals <1 year and between 1–2 years had progressively longer times to index medication add-ons. People with less severe diabetes may take longer to initiate their first therapy, and longer to get to their second. Clinical inertia, which refers to healthcare providers not initiating or intensifying therapy when indicated (Reach et al., 2017) could be a secondary explanation, as prescribers who are slow to prescribe initial therapy are likely to be slow to initiate further therapies. Potential contributors to clinical inertia include resistance to prescribing new medications and concerns about medication costs (Okemah et al., 2018). There are disadvantages of delaying treatment addition. Desai et al. found people taking metformin or a sulfonylurea with HbA_1C_ ≥7.0% (53 mmol/mol), who received an additional T2D therapy between 1–2 years were 22% less likely to achieve target glycaemic levels during the 7 years follow up compared with those who received one within 12 months (Desai et al., 2018). Finally, the median time between diagnosis and treatment initiation was longer for people initiating a sulfonylurea than metformin. Sulfonylurea initiators were older than metformin initiators and so this is consistent with a study by Zhang et al. who found that time to glucose-lowering medication initiation after T2D diagnosis was significantly longer for people aged ≥65 years than for those aged under 65 years (Zhang et al., 2012).

### Strengths and Limitations

Our study has several important strengths. Firstly, the NDSS data were nationally representative and included 80–90% of all people with T2D in Australia (Huo et al., 2018). Secondly, the data were linked to individual level dispensing data. Thirdly, this was the first study from Australia to investigate factors associated with treatment additions and switching. However, NDSS data were incomplete regarding clinical variables such as body mass index, smoking status, renal function or HbA_1C_. NDSS does not include information on ADEs of diabetes medications. We lacked comprehensive information on all patient demographics, lifestyle factors, co-morbid conditions and genetic factors. Genetic factors may modify the effect of sulfonylureas and thiazolidinediones which could, therefore, be associated with switching and addition (Mambiya et al., 2019). It is possible that add-ons were misclassified as switches if individuals were non-adherent to their index medication. However, our sensitivity analysis, which used longer grace periods, did not result in substantial changes to our results. Adherence to metformin and sulfonylureas may also be factors affecting the likelihood of add-on and switching. However, individuals with very poor adherence were censored due to apparent discontinuation of the treatment. Finally, we were unable to determine whether individuals used T2D medications as prescribed.

## Conclusion

Nearly one quarter of Australians who initiate treatment for T2D with metformin or sulfonylureas switch or receive additional treatment within 12-months, with those who initiate sulfonylureas more likely to switch or receive additional treatment than those who initiate metformin.

## Data Availability

The NDSS database is available upon application to the NDSS https://www.ndss.com.au/services/diabetes-research/access-to-ndss-data/.

## References

[B1] American Diabetes Association (2020). Addendum. 9. Pharmacologic Approaches to Glycemic Treatment: Standards of Medical Care in Diabetes-2020. Diabetes Care 2020;43(Suppl. 1):S98-S110. Diabetes Care 43 (1), 1979. 10.2337/dc20-ad08a 32503835

[B2] American Diabetes Association (2018). Older Adults: Standards of Medical Care in Diabetes. Diabetes Care 41 (1), 119–121. 10.2337/dc18-S011 29222382

[B3] Aschenbrenner D. S. (2016). The FDA Revises Restrictions on Metformin Use in Kidney Impairment. Am. J. Nurs. 116 (8), 22–23. 10.1097/01.naj.0000490173.76651.27 27466921

[B4] Banks E. Joshy G. Korda R. J. Stavreski B. Soga K. Egger S. (2019). Tobacco Smoking and Risk of 36 Cardiovascular Disease Subtypes: Fatal and Non-fatal Outcomes in a Large Prospective Australian Study. BMC Med. 17 (1), 128. 10.1186/s12916-019-1351-4 31266500 PMC6607519

[B5] Caughey G. E. Preiss A. K. Vitry A. I. Gilbert A. L. Ryan P. Shakib S. (2013). Does Antidepressant Medication Use Affect Persistence with Diabetes Medicines. Pharmacoepidemiol. Drug Saf. 22 (6), 615–622. 10.1002/pds.3424 23447430

[B6] Cornell S. (2017). Comparison of the Diabetes Guidelines from the ADA/EASD and the AACE/ACE. J. Am. Pharm. Assoc. (2003) 57 (2), 261–265. 10.1016/j.japh.2016.11.005 28065547

[B7] Desai U. Kirson N. Y. Kim J. Khunti K. King S. Trieschman E. (2018). Time to Treatment Intensification after Monotherapy Failure and its Association with Subsequent Glycemic Control Among 93,515 Patients with Type 2 Diabetes. Diabetes Care 41 (10), 2096–2104. 10.2337/dc17-0662 30131396

[B8] Erqou S. Lee C. C. Adler A. I. (2014). Statins and Glycaemic Control in Individuals with Diabetes: a Systematic Review and Meta-Analysis. Diabetologia 57 (12), 2444–2452. 10.1007/s00125-014-3374-x 25245638

[B9] Gerstein H. C. Swedberg K. Carlsson J. McMurray J. J. Michelson E. L. Olofsson B. (2008). The Hemoglobin A1C Level as a Progressive Risk Factor for Cardiovascular Death, Hospitalization for Heart Failure, or Death in Patients with Chronic Heart Failure: An Analysis of the Candesartan in Heart Failure: Assessment of Reduction in Mortality and Morbidity (CHARM) Program. Arch. Intern. Med. 168 (15), 1699–1704. 10.1001/archinte.168.15.1699 18695086

[B10] Górriz J. L. Soler M. J. Navarro-González J. F. García-Carro C. Puchades M. J. D’Marco L. (2020). GLP-1 Receptor Agonists and Diabetic Kidney Disease: a Call of Attention to Nephrologists. J. Clin. Med. 9 (4), 947. 10.3390/jcm9040947 PMC7231090 32235471

[B11] Grimes R. T. Bennett K. Tilson L. Usher C. Smith S. M. Henman M. C. (2015). Initial Therapy, Persistence and Regimen Change in a Cohort of Newly Treated Type 2 Diabetes Patients. Br. J. Clin. Pharmacol. 79 (6), 1000–1009. 10.1111/bcp.12573 25521800 PMC4456132

[B12] Hemmingsen B. Schroll J. B. Wetterslev J. Gluud C. Vaag A. Sonne D. P. (2014). Sulfonylurea versus Metformin Monotherapy in Patients with Type 2 Diabetes: a Cochrane Systematic Review and Meta-Analysis of Randomized Clinical Trials and Trial Sequential Analysis. Can. Med. Assoc. J. open 2 (3), E162–E175. 10.9778/cmajo.20130073 PMC4185978 25295236

[B13] Huo L. Magliano D. J. Rancière F. Harding J. L. Nanayakkara N. Shaw J. E. (2018). Impact of Age at Diagnosis and Duration of Type 2 Diabetes on Mortality in Australia 1997-2011. Diabetologia 61 (5), 1055–1063. 10.1007/s00125-018-4544-z 29473119

[B14] Khunti K. Gomes M. B. Pocock S. Shestakova M. V. Pintat S. Fenici P. (2018). Therapeutic Inertia in the Treatment of Hyperglycaemia in Patients with Type 2 Diabetes: a Systematic Review. Diabetes Obes. Metab. 20 (2), 427–437. 10.1111/dom.13088 28834075 PMC5813232

[B15] Koye D. N. Magliano D. J. Reid C. M. Pavkov M. E. Chadban S. J. McDonald S. P. (2019). Trends in Incidence of ESKD in People with Type 1 and Type 2 Diabetes in Australia, 2002-2013. Am. J. Kidney Dis. 73 (3), 300–308. 10.1053/j.ajkd.2018.10.005 30579709

[B16] Lee W.-C. Lee Y.-T. Li L.-C. Ng H.-Y. Kuo W.-H. Lin P.-T. (2018). The Number of Comorbidities Predicts Renal Outcomes in Patients with Stage 3-5 Chronic Kidney Disease. Jcm 7 (12), 493. 10.3390/jcm7120493 PMC6306906 30486496

[B17] Lycett D. Nichols L. Ryan R. Farley A. Roalfe A. Mohammed M. A. (2015). The Association between Smoking Cessation and Glycaemic Control in Patients with Type 2 Diabetes: A THIN Database Cohort Study. Lancet Diabetes Endocrinol. 3 (6), 423–430. 10.1016/S2213-8587(15)00082-0 25935880

[B18] Mambiya M. Shang M. Wang Y. Li Q. Liu S. Yang L. (2019). The Play of Genes and Non-genetic Factors on Type 2 Diabetes. Front. Public Health 7 (349), 349. 10.3389/fpubh.2019.00349 31803711 PMC6877736

[B19] Okemah J. Peng J. Quiñones M. (2018). Addressing Clinical Inertia in Type 2 Diabetes Mellitus: A Review. Adv. Ther. 35 (11), 1735–1745. 10.1007/s12325-018-0819-5 30374807 PMC6223992

[B20] Pantalone K. M. Misra-Hebert A. D. Hobbs T. M. Ji X. Kong S. X. Milinovich A. (2018). Clinical Inertia in Type 2 Diabetes Management: Evidence from a Large, Real-World Data Set. Diabetes Care 41, e113. 10.2337/dc18-0116 29678811

[B21] Paul S. K. Klein K. Thorsted B. L. Wolden M. L. Khunti K. (2015). Delay in Treatment Intensification Increases the Risks of Cardiovascular Events in Patients with Type 2 Diabetes. Cardiovasc. Diabetol. 14, 100–101. 10.1186/s12933-015-0260-x 26249018 PMC4528846

[B22] Pratt N. L. Kerr M. Barratt J. D. Kemp-Casey A. Kalisch Ellett L. M. Ramsay E. (2018). The Validity of the Rx-Risk Comorbidity index Using Medicines Mapped to the Anatomical Therapeutic Chemical (ATC) Classification System. BMJ Open 8 (4), e021122. 10.1136/bmjopen-2017-021122 PMC5905736 29654048

[B23] Reach G. Pechtner V. Gentilella R. Corcos A. Ceriello A. (2017). Clinical Inertia and its Impact on Treatment Intensification in People with Type 2 Diabetes Mellitus. Diabetes Metab. 43 (6), 501–511. 10.1016/j.diabet.2017.06.003 28754263

[B24] Royal Australian College of General Practitioners and Diabetes Australia (2014). General Practice Management of Type 2 Diabetes – 2014–15. Melbourne: The Royal Australian College of General Practitioners and Diabetes Australia, 47.

[B25] Royal Australian College of General Practitioners (2020). Management of Type 2 Diabetes: A Handbook for General Practice. Melbourne: Royal Australian College of General Practitioners.

[B26] Sola D. Rossi L. Schianca G. P. Maffioli P. Bigliocca M. Mella R. (2015). Sulfonylureas and Their Use in Clinical Practice. Arch. Med. Sci. 11 (4), 840–848. 10.5114/aoms.2015.53304 26322096 PMC4548036

[B27] Swedberg K. Rydén L. (2016). Treatment of Diabetes and Heart Failure: Joint Forces. Eur. Heart J. 37 (19), 1535–1537. 10.1093/eurheartj/ehw039 26908942

[B28] Thakker D. Nair S. Pagada A. Jamdade V. Malik A. (2016). Statin Use and the Risk of Developing Diabetes: a Network Meta-Analysis. Pharmacoepidemiol. Drug Saf. 25 (10), 1131–1149. 10.1002/pds.4020 27277934

[B29] Vitry A. I. Roughead E. E. Preiss A. K. Ryan P. Ramsay E. N. Gilbert A. L. (2010). Influence of Comorbidities on Therapeutic Progression of Diabetes Treatment in Australian Veterans: A Cohort Study. PLOS ONE 5 (11), e14024. 10.1371/journal.pone.0014024 21103337 PMC2984440

[B30] Wood S. J. Magliano D. J. Bell J. S. Shaw J. E. Keen C. S. Ilomäki J. (2020). Pharmacological Treatment Initiation for Type 2 Diabetes in Australia: Are the Guidelines Being Followed. Diabet Med. 37 (8), 1367–1373. 10.1111/dme.14149 31557346

[B31] World Health Organisation, (WHO) (2011). ATC: Structure and Principles. Oslo: WHO Collaborating Centre for Drug Statistics Methodology.

[B32] Zhang Q. Rajagopalan S. Marrett E. Davies M. J. Radican L. Engel S. S. (2012). Time to Treatment Initiation with Oral Antihyperglycaemic Therapy in US Patients with Newly Diagnosed Type 2 Diabetes. Diabetes Obes. Metab. 14 (2), 149–154. 10.1111/j.1463-1326.2011.01498.x 21952003

