# Supplementary material for: Treatment Dynamics in People Who Initiate Metformin or Sulfonylureas for Type 2 Diabetes: A National Cohort Study
Source: Front Pharmacol. 2021 Dec 14;12:794273. doi: 10.3389/fphar.2021.794273 (PMC8712936; doi:10.3389/fphar.2021.794273)
Supplement: Supplementary file 1 [file DataSheet1.docx]

## **Supplementary material**

**Appendix A:** RxRisk-V categories

| **Comorbidity or Condition** | **Anatomical Therapeutic Chemical Classification codes** |
| --- | --- |
| **Congestive heart failure** | (C03CA01-C03CC01) AND (C09AA01-C09AA16 OR C09CA01-C09CX99), C03DA04, C07AB07, C07AG02, C07AB12, C09DX04, C07AB02 ^a^ |
| **Depression** | N06AA01-N06AG02, N06AX03-N06AX11, N06AX13-N06AX26 |
| **Diabetes** | A10AA01-A10BX08 |
| **End stage renal disease** | B03XA01-B03XA03, V03AE02, V03AE03, V03AE05 |
| **Hyperlipidaemia** | C10AA01-C10BX12 |
| **Hypertension** | C02AB01-C02AC05, C02DB01-C02DB04, C03AA01-C03BA11, C03BB04, C03CA01-C03CC01, C03DA01-C03DA03, C03DB01-C03DB02, C03EA01-C03EA14, C09AA01-C09AA16, C09BA02-C09BA15, C09CA01-C09CA10, C09DA01-C09DA09 |
| **Nicotine dependence** | N07BA01-N07BA03, N06AX12 |

^a^ Metoprolol, (ATC code C07AB02); PBS item codes were used to identify the indication. PBS codes 8732N, 8733P, 8734Q and 8735R indicated congestive heart failure.

**Appendix B:** Sensitivity Analysis in Concessional Population with 2 Year Lookback Period

**Table B1.** Characteristics of Concession Population of Metformin and Sulfonylurea Initiators from the NDSS

|  | **Metformin initiators**  **(n=45,026)** | **Sulfonylurea initiators**  **(n=2,657)** | **Total**  **(n=47,683)** |
| --- | --- | --- | --- |
| **Age, years (mean±SD)** | 63.4±13.4 | 69.8±13.3 | 63.8±13.5 |
| **18-49** | 7,784 (17.3) | 234 (8.8) | 8,018 (16.8) |
| **50-74** | 29,071 (64.6) | 1,402 (52.8) | 30,473 (63.9) |
| **75-99** | 8,171 (18.1) | 1,021 (38.4) | 9,192 (19.3) |
| **Sex, female n(%) ^a^** | 22,240 (49.4) | 1,254 (47.2) | 23,494 (49.3) |
| **Comorbidity score (median [IQR]) ^b^** | 5 (3―6) | 6 (3―8) | 5 (3―6) |
| **Number of comorbidities ^b^** | | | |
| 0 | 1,098 (2.4) | 70 (2.6) | 1,168 (2.4) |
| 1-2 | 8,103 (18.0) | 363 (13.7) | 8,466 (17.8) |
| 3-4 | 12,950 (28.8) | 551 (20.7) | 13,501 (28.3) |
| 5+ | 22,875 (50.8) | 1,673 (63.0) | 24,548 (51.5) |
| **ARIA score** |  |  |  |
| **1. Major urban** | 27,617 (61.3) | 1,789 (67.3) | 29,406 (61.7) |
| **2. Inner regional** | 11,353 (25.2) | 503 (18.9) | 11,856 (24.9) |
| **3. Outer regional** | 5,341 (11.9) | 275 (10.4) | 5,616 (11.8) |
| **4. Remote** | 482 (1.1) | 50 (1.9) | 532 (1.1) |
| **5. Very remote** | 233 (0.5) | 40 (1.5) | 273 (0.6) |
| **SEIFA score (mean**±SD**)** | 2.7±1.4 | 2.8±1.4 | 2.7±1.4 |
| **1. Most disadvantaged** | 11,518 (25.6) | 683 (25.7) | 12,201 (25.6) |
| **2.** | 10,106 (22.4) | 507 (19.1) | 10,613 (22.3) |
| **3.** | 9,964 (22.1) | 568 (21.4) | 10,532 (22.1) |
| **4.** | 7,418 (16.5) | 473 (17.8) | 7,891 (16.5) |
| **5. Least disadvantaged** | 6,020 (13.4) | 426 (16.0) | 6,446 (13.5) |
| **Congestive heart failure** | 3,522 (7.8) | 487 (18.3) | 4,009 (8.4) |
| **Nicotine dependence** | 1,734 (3.9) | 56 (2.1) | 1,790 (3.8) |
| **Depression** | 13,185 (29.3) | 649 (24.4) | 13,834 (29.0) |
| **Systemic corticosteroids** | 6,722 (14.9) | 636 (23.9) | 7,358 (15.4) |
| **Antipsychotics** | 2,903 (6.4) | 143 (5.4) | 3,046 (6.4) |
| **Lipid-lowering medications** | 26,137 (58.0) | 1,521 (57.2) | 27,658 (58.0) |
| **Hypertension** | 25,017 (55.6) | 1,556 (58.6) | 26,573 (55.7) |
| **End stage renal disease** | 36 (0.1) | 127 (4.8) | 163 (0.3) |
| **Time between T2D diagnosis and index date (median [IQR]), years** | 0.1 (0.0―4.5) | 3.1 (0.0―9.1) | 0.1 (0.0―4.8) |
| **Time between T2D diagnosis and index date** | | | |
| **No delay** | 11,863 (26.3) | 471 (17.7) | 12,334 (25.9) |
| **<1 year** | 15,025 (33.4) | 621 (23.4) | 15,646 (32.8) |
| **1-2 years** | 1,935 (4.3) | 100 (3.8) | 2,035 (4.3) |
| **>2 years** | 16,203 (36.0) | 1,465 (55.1) | 17,668 (37.1) |
| **Aboriginal or Torres Strait Islander status** | | | |
| **Yes** | 1,496 (3.3) | 98 (3.7) | 1,594 (3.3) |
| **No** | 37,971 (84.3) | 2,284 (86.0) | 40,255 (84.4) |
| **Unspecified** | 5,559 (12.3) | 275 (10.4) | 5,834 (12.2) |

NDSS National Diabetes Services Scheme; T2D Type 2 Diabetes; ARIA Accessibility/ Remoteness Index of Australia; SEIFA Socio-Economic Indexes for Areas; SD Standard deviation; IQR Interquartile Range

**^a^** Unless otherwise stated, figures are quoted as n(%)

**^b^** A score of 1 was deducted from the total RxRisk-V score, as the whole cohort had T2D medications prescribed at baseline

**Table B2.** Factors Associated with Receiving Add-On Therapy or Treatment Switch Within One Year of Starting Metformin or Sulfonylurea in Concession Population

|  | **Metformin Add-On** | | **Metformin Switched** | | **Sulfonylurea Add-On** | | **Sulfonylurea Switched** | |
| --- | --- | --- | --- | --- | --- | --- | --- | --- |
|  | **HR** | **95%CI** | **HR** | **95%CI** | **HR** | **95%CI** | **HR** | **95%CI** |
| **Age, years** |  |  |  |  |  |  |  |  |
| **18-49** |  |  |  |  |  |  |  |  |
| **50-74** | 0.71 | (0.66―0.75) | 0.89 | (0.79―1.00) | 0.66 | (0.52―0.84) | 0.88 | (0.60―1.27) |
| **75-99** | 0.56 | (0.51―0.62) | 0.96 | (0.82―1.11) | 0.44 | (0.34―0.58) | 0.64 | (0.42―0.98) |
| **Sex, female** | 0.84 | (0.80―0.89) | 1.43 | (1.31―1.56) | 1.06 | (0.91―1.23) | 1.06 | (0.84―1.33) |
| **Number of comorbidities ^a^** | | | | | | | | |
| **0** |  |  |  |  |  |  |  |  |
| **1-2** | 0.72 | (0.62―0.82) | 0.91 | (0.69―1.21) | 0.74 | (0.50―1.08) | 0.62 | (0.35―1.10) |
| **3-4** | 0.66 | (0.57―0.76) | 0.97 | (0.73―1.29) | 0.52 | (0.35―0.78) | 0.50 | (0.28―0.90) |
| **5+** | 0.67 | (0.57―0.78) | 1.18 | (0.87―1.59) | 0.44 | (0.28―0.68) | 0.45 | (0.24―0.84) |
| **ARIA score** |  |  |  |  |  |  |  |  |
| **1. Major Urban** |  |  |  |  |  |  |  |  |
| **2. Inner Regional** | 0.95 | (0.90―1.02) | 1.04 | (0.94―1.15) | 0.86 | (0.69―1.07) | 1.31 | (0.99―1.75) |
| **3. Outer Regional** | 0.92 | (0.84―1.00) | 1.06 | (0.93―1.22) | 1.00 | (0.77―1.30) | 1.09 | (0.74―1.62) |
| **4/5 Remote and very remote** | 1.18 | (0.99―1.42) | 0.60 | (0.39―0.93) | 0.65 | (0.38―1.10) | 0.87 | (0.40―1.88) |
| **SEIFA index** |  |  |  |  |  |  |  |  |
| **1. Most Disadvantaged** |  |  |  |  |  |  |  |  |
| **2.** | 0.99 | (0.92―1.07) | 0.93 | (0.82―1.05) | 0.99 | (0.79―1.25) | 0.89 | (0.63―1.25) |
| **3.** | 0.98 | (0.91―1.05) | 0.93 | (0.82―1.05) | 0.95 | (0.76―1.20) | 1.04 | (0.75―1.44) |
| **4.** | 1.01 | (0.94―1.10) | 1.02 | (0.90―1.17) | 1.00 | (0.79―1.26) | 1.13 | (0.81―1.59) |
| **5. Least Disadvantaged** | 0.95 | (0.87―1.04) | 0.96 | (0.83―1.12) | 1.13 | (0.88―1.44) | 0.84 | (0.56―1.25) |
| **Congestive Heart Failure** | 1.30 | (1.18―1.43) | 1.24 | (1.07―1.44) | 1.30 | (1.04―1.62) | 0.71 | (0.50―1.03) |
| **Nicotine dependence** | 1.20 | (1.07―1.34) | 1.07 | (0.86―1.32) | 0.93 | (0.54―1.59) | 1.33 | (0.67―2.61) |
| **Depression** | 1.11 | (1.05―1.18) | 1.01 | (0.91―1.11) | 1.07 | (0.88―1.29) | 1.10 | (0.83―1.45) |
| **Systemic corticosteroids** | 1.16 | (1.08―1.25) | 1.19 | (1.06―1.33) | 1.00 | (0.82―1.23) | 1.23 | (0.92―1.64) |
| **Antipsychotics** | 1.20 | (1.09―1.32) | 0.96 | (0.80―1.14) | 1.60 | (1.16―2.20) | 0.99 | (0.57―1.71) |
| **Lipid-lowering medications** | 0.84 | (0.79―0.89) | 0.81 | (0.73―0.89) | 0.86 | (0.72―1.03) | 0.94 | (0.73―1.22) |
| **Hypertension** | 1.00 | (0.95―1.06) | 0.86 | (0.79―0.95) | 1.05 | (0.88―1.26) | 0.93 | (0.71―1.20) |
| **End stage renal disease** | 1.98 | (0.99―3.97) | 3.72 | (1.66―8.31) | 0.71 | (0.45―1.12) | 0.84 | (0.45―1.56) |
| **Time between T2D diagnosis and index date** | | | | | | | | |
| **No time** |  |  |  |  |  |  |  |  |
| **<1 year** | 0.86 | (0.80―0.91) | 0.91 | (0.82―1.02) | 0.84 | (0.68―1.03) | 0.84 | (0.61―1.16) |
| **1-2 years** | 0.73 | (0.63―0.84) | 0.86 | (0.68―1.09) | 0.47 | (0.29―0.76) | 0.40 | (0.18―0.87) |
| **>2 years** | 0.89 | (0.83―0.94) | 1.15 | (1.04―1.28) | 0.57 | (0.47―0.69) | 0.61 | (0.46―0.81) |
| **Aboriginal or Torres Strait Islander Status** | 1.24 | (1.10―1.40) | 0.90 | (0.70―1.16) | 0.85 | (0.55―1.33) | 0.69 | (0.33―1.42) |

T2D Type 2 Diabetes; ARIA Accessibility/ Remoteness Index of Australia; SEIFA Socio-Economic Indexes for Areas; CI confidence interval; HR adjusted hazard ratio

**^a^** A score of 1 was deducted from the total RxRisk-V score, as the whole cohort had T2D medications prescribed at baseline.
